# Supplementary material for: Profiling malaria infection among under-five children in the Democratic Republic of Congo
Source: PLoS One. 2021 May 6;16(5):e0250550. doi: 10.1371/journal.pone.0250550 (PMC8101767; doi:10.1371/journal.pone.0250550)
Supplement: Attachment — Submitted filename: 4 Profiling_Malaria_EminaR1_editor.docx [file pone.0250550.s002.docx]

**Profiling malaria infection among under-five children in the Democratic Republic of Congo**

Jacques B.O. Emina^* [1, 2]^; Henry V. Doctor ^[3]^; Yazoumé Yé ^[4]^

[1] Population and Health Research Institute, Kinshasa, Democratic Republic of Congo (DRC)

[2] Department of Population and Development Study, University of Kinshasa, DRC

[3] World Health Organization (WHO), Regional Office for the Eastern Mediterranean, Cairo, Egypt

[4] M&E Infectious Disease, ICF International, USA

*Corresponding author. Email: Jacques.emina@gmail.com

**Abstract**

**Introduction**

The number of health zones covered by the national malaria control program (PNLP) increased from 271 out 516 in 2009 to 516 in 2016. Malaria accounted for 38% of overall morbidity and 36% of overall mortality in DRC. This study aimed to identify malaria socioeconomic predictors among children aged 6-59 months in DRC; and describe socioeconomic profile of the most-at-risk children aged 6-59 months for malaria infection.

**Materials and Methods**

This study used data from the 2013 DRC Demographic and Health Survey (DHS). The sample included 8,547 children aged 6-59 months who got a malaria microscopy test (reading of thick-smear slide). Malaria infection status, the dependent variable, is a dummy variable characterized as a positive or negative test. The independent variables included child’ sex, age and living arrangement, mother’s education, household’ socio-economic variables; province of residence and type of place of residence. Statistical analyses relied on Pearson Chi-square, the Chi-square automatic interaction detector (CHAID) model and logistic regression.

**Results**

Of the 8,547 children considered, 25% had malaria infection. Of the ten variables analyzed, four were statistically associated with the prevalence of malaria infection in bivariate analysis and multivariate analysis (CHAID and logistic regression): child’s age, mother’ education, province and wealth index. Consistent with previous studies, the prevalence of malaria infection increases regularly with child’s age, and decreases significantly with mother’s education and the household wealth index. These findings suggest also that the prevalence of malaria infection is driven by interaction between environmental factors, socioeconomic characteristics and probably difference in the implementation of malaria programs across the country. The effect of maternal education on malaria infection was only significant among under-five children living in Ituri, Kasaï central, Haut Uele, Lomami, Nord Ubangi and Maniema provinces; while the effect of wealth index was significant in Mai-Ndombe, Tshopo and Haut Katanga provinces.

**Conclusion**

Findings from this study could be used for targeting malaria interventions in DRC. While malaria infection is common across DRC, the prevalence of children at high risk for malaria infection varies by province. These groups comprise children belonging to several socioeconomic categories (age, maternal education, wealth index, place of residence, etc.). Due to the multiplicity of areas that are linked to malaria transmission as well as high influence of province on malaria prevalence, designing province and multisectoral interventions could be the most effective strategy to achieve zero malaria infection in DRC.

**Key words**: Malaria, under-five children, Democratic Republic of Congo, Chi-square automatic interaction detector model.

**Background**

Malaria remains a threat to gains in health and development ^[1-5]^ though the number of deaths due to this disease has been halved since 2000 ^[3-4, 6]^. One component of the global technical strategy (GTG) for malaria 2016–2030 in the African region is to accelerate efforts towards elimination and attainment of malaria-free status by 2030 ^[7]^.

Accounting for 11% of the global malaria burden, malaria is a major public health concern in the Democratic Republic of Congo (DRC) ^[8]^. In 1998, DRC created a national malarial control program [Programme National de Lutte contre le Paludisme (PNLP)]. The main objective of this program was to reduce malaria mortality by 50% and malaria morbidity by 25% by 2015. Its activities consist of the following interventions: distribution of insecticide-treated mosquito nets (ITNs), promotion of indoor residual spraying (IRS), promotion and implementation of intermittent preventive treatment in pregnancy (IPTp), promotion of rapid diagnostic tests (RDTs), and implementation of Community and Mother Case Management with artemisinin combination therapies (ACTs) ^[9-10]^.

PNLP has intensified implementation of malaria control strategies over the last 15 years ^[9]^. The number of health zones covered by the national malaria control program (PNLP) increased from 271 out of 516 health zones in 2009 to 516 (all health zones) in 2016 ^[9]^. In addition, the NMCP established a network of 11 sentinel sites for an integrated surveillance of priority diseases, including malaria in 2003, with an expansion to the 26 new provinces in 2016 ^[9]^. Despite this progress, malaria accounted for 38% of overall morbidity and 36% of overall mortality ^[4,8-10]^ in DRC. In 2018, the country reported 15 million malaria cases and about 27,458 malaria deaths ^[8]^. A large majority of malaria cases and rapid progression to death occur in young children ^[8-10]^. Therefore, the gap between the current state of malaria and the WHO goals of zero malaria case by 2030 remains important for the DRC.

Although during the last three decades interest in understanding factors associated with malaria among under-five children has increased in sub-Saharan Africa, less is known about the socioeconomic profile of under-five children at higher risk of malaria infection in DRC. Published studies on malaria infection among under-five children could be divided into three groups. Some studies have analyzed trends in the prevalence of malaria infection among under-five children at the national and sub-national (province, district and health zone levels) ^[11-12^]. Others have described socioeconomic factors associated with malaria morbidity and mortality among patients attending health facilities ^[13-15]^. Some other studies have analyzed individual and community socioeconomic factors associated with malaria infection ^[16-18]^. This situation is due to data limitations. The 2013 Democratic Republic of Congo Demographic and Health Survey is the first national survey which collected data on malaria status, individual characteristics, maternal variables and households’ characteristics. Using chi-square and regression models, these studies did not include automatically interactions between variables. The most-at-risk groups for malaria infection could be a result of different interactions between malaria risk factors. For instance, existing literature revealed that children living in poorest households, children of the less educated mothers, those living in rural areas, and children aged above 23 months, had greater risk of malaria infection [13-18]. However, a child could belong to all these risk categories, others to the least risk groups or to some high risk and to least risk groups at the same time ^[8]^.

Against this backdrop, this study aims to identify socioeconomic predictors of malaria and describe socioeconomic profile of malaria prevalence among under-five children in the DRC, considered as one of most-at-risk groups affected by malaria using chi-square, logistic regression and Chi-square Automatic Interaction Detector (CHAID). Findings from such study will allow designing targeted interventions and evidence-based prevention programs as well as optimize coverage, reduce costs and lower the number of new infections given the high cost of interventions, 194 million USD in 2013 [9-10].

**Malaria risk factors and the most-at-risk for malaria**

People are at risk of acquiring malaria infection due to factors related to environment, demographic, socio-economic status and exposure to prevention interventions ^[6, 11-18]^.

Figure 1 presents selected risk factors and categories under higher and lower risk of malaria using WHO classification of malaria epidemiology ^[19]^.

[Figure 1 about here]

Environmental and ecological factors, including the distances from household and the nearest water-body, altitudes, temperature and rainfall determine malaria transmission zone: high transmission zone (Mesoendemic, Hyperendemic and Holoendemic) or low transmission zone (Hypoendemic malaria) ^[19-20]^. In this study, province of residence has been used as proxy of geographical location.

Considering socioeconomic factors, children living in poor households, those whose mothers are less educated and children living in rural areas are more likely to suffer from malaria ^[13-26]^. Individuals belong to several categories, which in turn might belong to different malaria clusters (high risk or low risk).

**Data and Methods**

*Variables*

The dependent variable for this analysis is the malaria infection status defined as a positive or negative malaria test. The independent variables include 10 variables grouped into three major types including: (1) child variables (sex, age, living arrangement, whether slept under an Insecticide-Treated Nets (ITN) the night preceding data collection); and (2) mother’s education and household’s characteristics (sex of the head of household, age of the head of household, wealth index); (3) and contextual factors, including province and place of residence. The choice of these variables is guided by the literature on factors associated with malaria infection ^[6, 13-26].^

*Data sources*

This study used data from the 2013 DRC Health and Demographic Survey (DRC-DHS). The survey used a two-stage stratified-cluster sampling design based on the sampling frame of the 1984 Population and Housing Census, which has been partially updated several times by administrative censuses and in the context of the presidential and legislative elections of 2011. The final survey unit chosen is the cluster (district or village) and, in total, 540 clusters were drawn. The selection of clusters known as Primary Sampling Units (PSUs) was made in the first stage. The second stage involves selection of households from each cluster. Stratification in the first stage was achieved by grouping the 11 provinces into urban and rural areas. Furthermore, PSUs in the provinces with very small population were selected with equal size allocation.

The DHS data offer a unique opportunity to profile malaria infection in the country due to the paucity of routine data which are associated with unknown denominator and selection^[1]^ bias and lack of individuals and households’ characteristics considered as predictors of the epidemy. The DHS incorporated five biomarker tests which include malaria testing. Malaria testing was carried out among children aged 6-59 months in half of the 18,360 selected households. Malaria testing using microscopy was carried out among children aged 6-59 months in half of selected households. Using a finger (or heel) prick, a drop of blood was collected on a slide to prepare a thick film. After drying, the slides were stored in special boxes with desiccants and humidity controllers. These dishes were transferred regularly to the NRL for the detection of hematozoa by microscopy, which was carried out regularly. All health technicians were trained to perform finger (or heel) pricks in the field according to the manufacturers’ instructions. A total of 8,547 children aged 6-59 months were tested for malaria. The survey report provides more details on the sampling and microscopy process ^[26]^.

*Statistical analyses*

Statistical analyses relied on Pearson’s χ2, using the Chi-square Automatic Interaction Detector (CHAID) decision-tree algorithm implemented in SPSS V.21, and logistic regression. Pearson’s χ2 was performed to identify associations between the malaria infection (positive or negative) and independent variables, including socio-economic and demographic characteristics. The study applied the nominal CHAID model to identify the most significant determinants of malaria infection among under-five children; and to describe the characteristics of the most-at-risk children for malaria infection considering interactions between predictors ^[27-31]^. The model operates sequentially by recursively splitting under-five children into separate and distinct segments called nodes. The variation of the prevalence of malaria infection is minimized within each node and is maximized between nodes. After the initial splitting of the population (under-five children who received malaria test) into different nodes based on the most significant predictor, the model repeats the process on each of the nodes until no significant predictors remain or when number of observations in the node does not allow further partitions. Ideally the minimum number of cases is estimated at 50 cases for child nodes though one can lower this minimum number of cases ^[27-30]^.

CHAID displays outcomes in hierarchical tree-structured form, in which the root is the population, under-five children who received malaria test in this case. The root node, ‘Node 0’ or ‘initial Segment’, is the outcome variable and subsequent levels include parent node and child node. Parent node is the upper node compared with nodes on the subsequent (lower) level, whereas any sub-node of a given node is called a child node. Sibling nodes are nodes on the same hierarchical level under the same parent node. Ancestors node comprise all nodes higher than a given node in the same lineage; whereas all nodes below the given node are called descendants. The terminal nodes consist of any node, which does not have child nodes. They are the last categories of the CHAID tree. Findings include also a table with five major columns describing each terminal node regarding their content, population size, number suffering from malaria infection, the prevalence of malaria infection in each terminal node ^[27-31]^. The analysis is focused on column 4, prevalence of malaria infection in each terminal node (category).

The study also employed logistic regression to identify predictors of malaria infection among children aged less than five years. It consists of comparing the proportions using the logarithms of the odds ratio (log-odds). The model estimates, for each selected category, the parameter ß (the ratio between the logit of a selected group and that of the reference group) and calculates the odds ratios while specifying their significance level (95% in this case) ^[31-32]^. If the odds ratio is equal to one, there is no difference between the considered group and the reference group regarding the risk of suffering from malaria. If the odds ratio is less than unity, children belonging to the considered group is less likely to suffer from malaria infection compared to their counterpart belonging to the reference group. By contrast, if the odds ratio is greater than one, children belonging to the selected group are more likely to suffer from malaria infection than their counterpart belonging to the reference group ^[30-32]^. However, the logistic regression model fails to incorporate non-monotonic relationships. Furthermore, it does not automatically detect interactions between segments or categories of independent variables. Significant differences have been established at p<0.05.

*Data analysis strategies*

We weighted data (CHAID in SPSS) and applied the SVY (Logistic regression in STATA 15) to account for the complex design of the household survey. Missing values were treated as a separate category. For instance, “Do not know” category for maternal education included children whose mother’s education was missing.

*Ethical considerations*

The DHS questionnaire, procedures and the testing protocol undergo a host country ethical review (the DRC school of public health ethical review committee) as well as an Institutional Review Board (IRB) at ICF Macro. Furthermore, participation in individual survey and in malaria testing is voluntary. Parents should sign the consent form before interview and before children’ blood collection.

Interviews and biomarker testing were performed as privately as possible. Results of interviews and biomarker testing were strictly confidential. Only the DHS research team (interviewers, health specialists, editors, and supervisors) were allowed to access the data essentially for communications. Each respondent's interview and biomarker data files were identified only by a series of numbers. The questionnaire cover sheets containing identifier numbers were destroyed after data processing.

**Results**

*Participants*

Table 1 shows the distribution of the study population by selected background characteristics. Of the total 8,547 children aged 6-59 months who were tested for malaria, 50% were female.

The distribution of the sample by age shows that 11% of the population was aged 6-11 months. The average age of the sample was estimated at 32.4 months (standard deviation 15.7). Children living with both parents constituted about 64% of the sample. A majority of participants lived in rural areas (71%) and in households headed by males (77%). Half of the population (50%) were living in households headed by people aged 25-39 years, whereas 4% of children were living in household headed by people aged 65 years or above. By province, the sample size varies from 2.5% (Lualaba) to 5.7% (Sud Kivu). About half of children tested (53%) slept under Insecticide-treated bed nets (ITN) the night preceding the survey. Regarding the household wealth index, 27% of children were living in poorest households, whereas 13% were living richest households.

Table 1 – Descriptive characteristics of children aged 6-59 months who had a malaria test, Democratic Republic of Congo Demographic Health Survey, 2013

| **Background variables** | **%** | **N** |  | **Background variables** | **%** | **N** |
| --- | --- | --- | --- | --- | --- | --- |
| **Sex of child** |  |  |  | **Province** |  |  |
| Male | 49.8 | 4,258 |  | Kinshasa | 5.3 | 453 |
| Female | 50.2 | 4,289 |  | Kwango | 4.4 | 379 |
| **Child’s age group (months)** |  |  |  | Kwilu | 5.0 | 424 |
| 6-11 | 11.0 | 943 |  | Mai-Ndombe | 3.9 | 331 |
| 12-23 | 22.0 | 1,882 |  | Kongo central | 4.5 | 386 |
| 24-35 | 22.2 | 1,894 |  | Equateur | 2.9 | 245 |
| 36-47 | 22.7 | 1,943 |  | Mongala | 3.5 | 299 |
| 48-59 | 22.1 | 1,885 |  | Nord-ubangi | 3.0 | 255 |
| **Living arrangement** |  |  |  | Sud-Ubangi | 3.7 | 314 |
| Not living with mother | 9.0 | 770 |  | Tshuapa | 2.8 | 239 |
| Living with mother only | 26.5 | 2,266 |  | Kasai | 4.4 | 374 |
| Living with both parents | 64.5 | 5,511 |  | Kasai-central | 4.3 | 368 |
| **Mother's education** |  |  |  | Kasai-oriental | 3.6 | 310 |
| None | 19.8 | 1,689 |  | Lomami | 4.7 | 405 |
| Primary | 40.8 | 3,490 |  | Sankuru | 3.5 | 299 |
| Secondary and above | 30.4 | 2,597 |  | Haut-katanga | 3.5 | 295 |
| Don’t Know | 9.0 | 771 |  | Haut-lomami | 3.5 | 295 |
| **Wealth index** |  |  |  | Lualaba | 2.5 | 209 |
| Poorest | 27.0 | 2,305 |  | Tanganyka | 3.3 | 279 |
| Poorer | 22.4 | 1,915 |  | Maniema | 4.9 | 415 |
| Middle | 20.2 | 1,730 |  | Nord-kivu | 5.7 | 483 |
| Richer | 17.6 | 1,503 |  | Bas-Uele | 2.6 | 225 |
| Richest | 12.8 | 1,094 |  | Haut-uele | 2.6 | 223 |
| **Sex of the head of household** |  |  |  | Ituri | 3.4 | 291 |
| Male | 77.3 | 6,605 |  | Tshopo | 3.1 | 266 |
| Female | 22.7 | 1,942 |  | Sud-Kivu | 5.7 | 485 |
| **Age of the head of household** |  |  |  | **Place of residence** |  |  |
| <25 | 5.8 | 498 |  | Capital and large cities | 12.8 | 1,094 |
| 25-34 | 34.6 | 4,321 |  | Small cities and town | 16.5 | 1,409 |
| 35-44 | 29.8 | 2,042 |  | Countryside | 70.7 | 6,044 |
| 45-64 | 25.6 | 948 |  | **Used ITN last night** |  |  |
| 65+ | 4.2 | 738 |  | No | 47.1 | 4,022 |
|  |  |  |  | Yes | 52.9 | 4,525 |
| Total | **100.0** | **8,547** |  |  |  |  |

Note: ITN - insecticide-treated nets

*Factors associated with malaria prevalence: Findings from bivariate analysis*

Overall, out of 8,547 children considered, 25% (95% confidence interval: 24.3%-26.2%) had malaria infection. Table 2 reports the prevalence of malaria infection among under-five children in DRC by selected background characteristics. Of the 10 independent variables included in the study, six were statistically significantly associated with malaria infection status. Child’ sex, sex and age of the head of household were not statistically associated with the likelihood of suffering from malaria infection.

The prevalence of malaria infection regularly increased with age. The percentage of children suffering from malaria was estimated at 14% among those age 6-11 months and 31% among children age 48-59 months. The prevalence of malaria infection was low among children living with their mothers alone (23%) or with the two parents (25%) compared to those others (31%). Table 2 also shows significant negative association between maternal education and malaria infection among under-five children: Malaria prevalence was higher among children whose mother did not attend school and lower among children whose mother had secondary or higher education (29% versus 17%).

Table 2 Malaria prevalence by selected socioeconomic characteristics

| **Variables** | % | N | Chi-sq | P-value |  | **Variables** | % | N | Chi-sq | P-value |
| --- | --- | --- | --- | --- | --- | --- | --- | --- | --- | --- |
| **Sex of child** |  |  |  |  |  | **Place of residence** |  |  |  |  |
| Male | 25.7 | 4,258 | 1.085 | 0.298 |  | Capital&large cities | 17.6 | 1,094 |  |  |
| Female | 24.7 | 4,289 |  |  |  | Small cities&Town | 24.6 | 1,409 | 41.177 | <0.001 |
| **Child’s age group** |  |  |  |  |  | Countryside | 26.8 | 6,044 |  |  |
| 6-11 | 14.5 | 943 |  |  |  | **Province** |  |  |  |  |
| 12-23 | 19.1 | 1,882 | 159.688 | <0.001 |  | Kinshasa | 16.6 | 453 |  |  |
| 24-35 | 25.2 | 1,894 |  |  |  | Kwango | 9.0 | 379 |  |  |
| 36-47 | 30.4 | 1,943 |  |  |  | Kwilu | 8.0 | 424 |  |  |
| 48-59 | 31.4 | 1,885 |  |  |  | Mai-ndombe | 29.3 | 331 |  |  |
| **Living arrang.** |  |  |  |  |  | Kongo central | 26.4 | 386 |  |  |
| Not living with mother | 30.8 | 770 |  |  |  | Equateur | 17.6 | 245 |  |  |
| Living with mother only | 23.6 | 2,266 | 15.927 | <0.001 |  | Mongala | 15.4 | 299 |  |  |
| Living with both parents | 25.1 | 5,511 |  |  |  | Nord-ubangi | 36.9 | 255 |  |  |
| **Mother's Education** |  |  |  |  |  | Sud-ubangi | 20.4 | 314 |  |  |
| None | 29.0 | 1,689 |  |  |  | Tshuapa | 15.9 | 239 |  |  |
| Primary | 28.3 | 3,490 | 135.171 | <0.001 |  | Kasai | 30.0 | 374 |  |  |
| Secondary & above | 17.0 | 2,597 |  |  |  | Kasai-central | 38.0 | 368 | 611.068 | <0.001 |
| Don’t know | 30.7 | 771 |  |  |  | Kasai-oriental | 27.7 | 310 |  |  |
| **Sex of the hh** |  |  |  |  |  | Lomami | 38.3 | 405 |  |  |
| Male | 25.6 | 6,605 | 1.848 | 0.174 |  | Sankuru | 19.7 | 299 |  |  |
| Female | 24.1 | 1,942 |  |  |  | Haut-katanga | 26.1 | 295 |  |  |
| **Age of the hh** |  |  |  |  |  | Haut-lomami | 23.4 | 295 |  |  |
| <25 | 24.5 | 498 |  |  |  | Lualaba | 41.6 | 209 |  |  |
| 25-34 | 24.9 | 4,321 |  |  |  | Tanganyka | 50.2 | 279 |  |  |
| 35-44 | 23.8 | 2,042 | 8.880 | 0.064 |  | Maniema | 35.7 | 415 |  |  |
| 45-64 | 27.5 | 948 |  |  |  | Nord-kivu | 8.1 | 483 |  |  |
| 65&+ | 25.1 | 738 |  |  |  | Bas-uele | 43.6 | 225 |  |  |
| **Wealth Index** |  |  |  |  |  | Haut-uele | 35.0 | 223 |  |  |
| Poorest | 26.9 | 2,305 |  |  |  | Ituri | 37.1 | 291 |  |  |
| Poorer | 29.7 | 1,915 |  |  |  | Tshopo | 26.7 | 266 |  |  |
| Middle | 27.2 | 1,730 | 132.722 | <0.001 |  | Sud-kivu | 12.8 | 485 |  |  |
| Richer | 24.4 | 1,503 |  |  |  | **Used itn last night** |  |  |  |  |
| Richest | 11.8 | 1,094 |  |  |  | No | 28.7 | 4,022 | 49.108 | <0.001 |
| **Total** | 25.2 | **8,547** |  |  |  | Yes | 22.1 | 4,525 |  |  |

The prevalence of malaria infection was higher in rural areas (27%) and small cities and towns (25%), than in large cities, including Kinshasa, the capital city (18%). The proportion of children suffering from malaria was lower in the richest households (12%) compared to those living to all other households (from poorest to richer). Findings also show low prevalence of malaria infection (22%) among children who slept under ITN compared to those who didn’t. (29%). The malaria endemicity shows regional heterogeneity with a higher prevalence (50%) observed in the Tanganyika province. Children living in Kwango (9%), Kwilu (8%) and Nord Kivu (8%) had the lowest prevalence of malaria infection.

*Socioeconomic predictors of malaria: Findings from CHAID model*

Box 1 shows summary information on the specifications used to build the final CHAID model. Ten independent variables were examined among which five were statistically significant in the final model.

| Box 1- Malaria prevalence among under-five children: Summary of CHAID model | | |
| --- | --- | --- |
| *Model components* | *Model specification* | *Results* |
| Dependent variable | Parasitemia (via microscopy) in children 6-59 months | 25% |
| Independent variables | Child’ sex, Child age, Children's living arrangement, ITN used the night before the survey, Place of residence, Mother’s education, Age of the head of household, sex of head of household, Province, Wealth index | Province, Child age, Place of residence, Mother education, Wealth index |
| Maximum tree depth | 3 | 3 |
| Minimum number of children in parent node | 100 | 100 |
| Minimum number of children in child node | 50 | 50 |
| Number of nodes | Na | 42 |
| Number of terminal nodes | Na | 26 |

The CHAID tree diagram depicted in Figure 2a shows that the province of residence (χ2=603.06, p<0.001) is the best predictor of malaria infection. Figures 2a-2e and Table 3 report predictors of malaria infection among under-five children in DRC by province.

Depending on province, the main predictors include child’s age, wealth index, place of residence and maternal education.

Table 3 – Socioeconomic predictors of malaria infection among under-five children by province, DRC Demographic and Health Survey, 2013

| **Province** | **First predictor** |  | **Second predictor** |
| --- | --- | --- | --- |
| Tshuapa, Equateur, Kinshasa, Sud Kivu, Mongala | Child’s age  (χ2=31.82, p<0.001) |  | Place of residence (χ2 depends on child’s age and province) |
| Kwilu, Nord Kivu, Kwango | Place of residence  (χ2=14.62, p<0.001) |  | Child’s age (χ2 depends on place of residence and province) |
| Ituri, Kasai-Central, Haut-Uele, Lomami, Nord -Ubangi, Maniema | Child’s age  (χ2=53.15, p<0.001) |  | Maternal education (χ2 depends on maternal education category and province) |
| Tanganyika | - |  | - |
| Mai-Ndombe; Tshopo; Haut-Katanga; Kongo Central; Kasai, Kasai Oriental | Wealth index  (χ2=111.16, p<0.001) |  | Child’s age (χ2 depends on wealth index and province) |
| Haut Lomami, Sankuru, Sud-Ubangi | Child’s age  χ2=19.606, p<0.001 |  | - |
| Lualaba, Bas-Uele | Place of residence (χ2=12.57, p<0.001) |  | - |

No subsequent malaria infection predictor was identified in Tanganyika. In Haut-Lomami, Sankuru and Sud-Ubangi, child’s age is the only significant predictor of malaria prevalence (χ2=19.61, p<0.001). Likewise, in Lualaba, Bas-Uele, place of residence (χ2=12.57, p<0.001) is the only significant predictor of malaria infection among under-five children.

*Malaria infection among under five children: Risk groups*

The CHAID model splits participants into 26 homogeneous groups or terminal nodes regarding the prevalence of malaria infection. Figures 2a-2e depicts the process of homogeneous groups creation, including variables comprised in each category. Table 4 describes these groups by their size (columns A and B), number of children with malaria infection (column C), the share in children with malaria infection (column E), and proportionality of the share in malaria epidemic compared to the demographic weight (column F). The 26 homogenous subgroups could be grouped into four major clusters (the third cluster includes two sub-clusters) consistent with WHO classification of malaria epidemiology ^[19].^ Table 4 reports the characteristics of each group.

Table 4 – Chi-square automatic interaction detector risk groups

| **Node description** | | Node size | | Tested + | | Prevalence | Index^[f]^ |
| --- | --- | --- | --- | --- | --- | --- | --- |
|  | | N^[a]^ | %^[b]^ | N^[c]^ | %^[d]^ | (%)^[e]^ |  |
| ***Cluster 1*** | | **493** | **5.8** | **263** | **12.2** | **53.3** | **211.5** |
| Poorer in Haut Katanga and Kasaï Oriental | | 73 | 0.9 | 47 | 2.2 | 64.4 | 255.2 |
| Living in rural area in Lualaba | | 141 | 1.6 | 76 | 3.5 | 53.9 | 213.7 |
| Living in Tanganyika | | 279 | 3.3 | 140 | 6.5 | 50.2 | 198.9 |
| ***Cluster 2*** | | ***6186*** | ***72.4*** | ***1761*** | ***81.7*** | ***28.5*** | ***112.9*** |
| Age 36-59 in Ituri, Kasaï central, Haut Uele, Lomami, Nord Ubangi & Maniema | | 908 | 10.6 | 407 | 18.9 | 44.8 | 177.7 |
| Living in rural area in Bas Uele | | 183 | 2.1 | 78 | 3.6 | 42.6 | 169.0 |
| Age 36-59 in poorest/middle/richer quintiles in Mai-Ndombe, Tshopo, Haut Katanga, Kongo central, Kasaï, Kasaï oriental | | 532 | 6.2 | 201 | 9.3 | 37.8 | 149.8 |
| Age 12-35 from mother with primary education living in Ituri, Kasaï central, Haut Uele, Lomami, Nord Ubangi & Maniema | | 436 | 5.1 | 159 | 7.4 | 36.5 | 144.6 |
| Living in poorer quintile in Mai-Ndombe, Tshopo, Kongo central, Kasaï | | 313 | 3.7 | 111 | 5.1 | 35.5 | 140.6 |
| Age 36-59 living in small cities/Town in Tshuapa, Equateur, Sud Kivu, Mongala | | 73 | 0.9 | 25 | 1.2 | 34.2 | 135.8 |
| Age 6-11 in Ituri, Kasaï central, Haut Uele, Lomami, Nord Ubangi & Maniema mothers never no or primary education | | 129 | 1.5 | 40 | 1.9 | 31.0 | 122.9 |
| Age 24-35 living in poorest/middle/richer quintiles in Mai-Ndombe, Tshopo, Haut Katanga, Kongo central, Kasaï, Kasaï oriental | | 252 | 2.9 | 73 | 3.4 | 29.0 | 114.8 |
| Living in small cities/Town (Bas Uele and Lualaba) | | 110 | 1.3 | 31 | 1.4 | 28.2 | 111.7 |
| Age 12-35 from never educated mother or mother with secondary education or highest living in Ituri, Kasaï central, Haut Uele, Lomami, Nord Ubangi & Maniema | | 394 | 4.6 | 109 | 5.1 | 27.7 | 109.7 |
| Age 24-59 living in Haut Lomami, Sankuru, Sud-Ubangi | | 607 | 7.1 | 154 | 7.1 | 25.4 | 100.6 |
| Age 24-35 living in Kinshasa, Equateur | | 156 | 1.8 | 34 | 1.6 | 21.8 | 86.4 |
| Age 6-23 in poorest/middle/richer quintiles in Mai-Ndombe, Tshopo, Haut Katanga, Kongo central, Kasaï, Kasaï oriental | | 427 | 5.0 | 86 | 4.0 | 20.1 | 79.8 |
| Age 36-59 living in Capital/large cities or rural areas in Tshuapa, Equateur, Kinshasa, Sud Kivu, Mongala | | 681 | 8.0 | 128 | 5.9 | 18.8 | 74.5 |
| Living in richest quintile in Tshopo, Kasaï, Kasaï oriental | | 128 | 1.5 | 19 | 0.9 | 14.8 | 58.8 |
| Living in large cities/small cities and town in Kwilu, Nord Kivu and Kwango | | 320 | 3.7 | 43 | 2.0 | 13.4 | 53.3 |
| Age 6-23 living in Haut Lomami, Sankuru, Sud-Ubangi | | 301 | 3.5 | 38 | 1.8 | 12.6 | 50.0 |
| Age 24-35 living in Tshuapa, Sud Kivu and Mongala | | 236 | 2.8 | 25 | 1.2 | 10.6 | 42.0 |
| ***Cluster 3*** | | ***1868*** | ***21.9*** | ***132*** | ***6.1*** | ***7.1*** | ***28.0*** |
| Age 6-23, living in Tshuapa, Equateur, Kinshasa, Sud Kivu and Mongala | | 575 | 6.7 | 52 | 2.4 | 9.0 | 35.9 |
| Age 6-11 living Ituri, Kasaï central, Haut Uele, Lomami, Nord Ubangi & Maniema, mothers with secondary education or highest/ mother’s education unknown | | 90 | 1.1 | 8 | 0.4 | 8.9 | 35.2 |
| Age 24-59 living rural areas in Kwilu, Nord Kivu and Kwango | | 658 | 7.7 | 58 | 2.7 | 8.8 | 34.9 |
| Living in richest quintile in Mai-Ndombe, Haut Katanga, Kongo central | | 237 | 2.8 | 8 | 0.4 | 3.4 | 13.4 |
| Age 6-23 living rural areas in Kwilu, Nord Kivu and Kwango | | 308 | 3.6 | 6 | 0.3 | 1.9 | 7.7 |
| ***Overall*** | | ***8547*** | ***100.0*** | ***2156*** | ***100.0*** | ***25.2*** | ***100.0*** |
| Notes: N number of children; ////  [^a^] Number of children who got malaria test;  [^b^] Demographic size in percentage = ([^a^] /Σ[^a^]) × 100;  [^c^] Number of children tested positive | [^d^] Demographic size in percentage among children tested positive = ([^c^] /Σ[^c^]) × 100;  [^e^] Prevalence of malaria in each group = ([^c^] /Σ[^a^]) × 100;  [^f^] Node index (Proportionality index) = [([^c^] /Σ[^c^]) /([^a^] /Σ[^a^])] × 100 | | | | | | |

[Figures 2a about here]

[Figures 2b about here]

[Figures 2c about here]

[Figures 2d about here]

[Figures 2e about here]

*Cluster 1 – Children living in Poor households in Haut-Katanga and Kasaï Oriental*

Children belonging to this cluster represent 1% of participants and 2% of children tested positive to malaria infection, yielding to an index of 255%. Malaria prevalence was estimated at 64% in this cluster.

*Cluster 2 – Children living in Tanganyika and in rural Lualaba*

The prevalence of malaria infection was estimated at 51.4% among children living in the Tanganyika province and the rural area of Lualaba. This cluster accounts for 4% of participants and 10% of children with malaria infection, yielding to an index of 204%. Like the previous cluster, it is located in the southern belt of DRC.

*Cluster 3 –Mixed socioeconomic categories and different provinces*

Cluster 3 includes the larger group of children (72.4% of children who got a malaria test). Malaria prevalence was estimated at 28.5%, ranged from 10.6% (children aged 24-35 living in Tshuapa, Sud, Kivu and Mongala) to 45% (children aged 36-59 in Ituri, Kasaï central, Haut Uele, Lomami, Nord Ubangi & Maniema). Children belonging to this cluster represent 82% of children suffering from malaria infection. They live in 25 provinces out of 26. This cluster includes children belonging to all age groups and socioeconomic characteristics (poorest - richest, living in rural and urban areas, children whose mothers never attended school, those of mothers with primary to secondary education).

*Cluster 4 – Young children or living in high socioeconomic strata in 20 provinces*

This cluster includes 22% of children who got malaria test. The prevalence of malaria was estimated at 7%, and accounts for 6% of all children suffering from malaria. This cluster includes five subgroups: (1) children aged 6-23 months and living in Tshuapa, Equateur, Kinshasa, Sud Kivu and Mongala; (2) children aged 6-11 months, living Ituri, Kasaï central, Haut-Uele, Lomami, Nord Ubangi and Maniema whose mothers have secondary education and highest or mother’s education is unknown; (3) children aged 24-59 months living rural areas in Kwilu, Nord Kivu and Kwango; (4) children living in richest quintile in Mai-Ndombe, Haut Katanga and Kongo central; (5) children aged 6-23 months living rural areas in Kwilu, Nord Kivu and Kwango.

*Socioeconomic predictors of malaria: Findings from logistic regression model*

Of the nine variables included in the logistic regression model (Table 5), five are statistically associated with the prevalence of malaria infection among children aged less than five years: child’ age, sleeping under a mosquito net, maternal education, household wealth index and province of residence. The risk of suffering from malaria infection increases significantly with child’ age. Compared to children aged 6-11 months, those aged 12-23 have 1.7 times more risk of suffering from malaria [95% Confidence Interval (CI)=1.24-2.36]. This risk is estimated at 2.6 [95% Confidence Interval (CI)=2.02-3.54]; 3.3 [95% Confidence Interval (CI)=2.53-4.46] and 3.4 times 6 [95% Confidence Interval (CI)=2.53-4.62] for children aged 24-35, 36-47 and 48-59 months respectively.

The likelihood of suffering from malaria infection for an under-five child is low among children living in least poor households. The risk of suffering from malaria infection is 31% lower [odds-ratio=0.61; 95% Confidence Interval (CI)=0.50-0.97] among children living in the richer households, and about 81% lower [odds-ratio=0.19; 95% Confidence Interval (CI)=0.09-0.37] among children living in the richest household compared to children who live in the poorest households. Considering maternal education, children whose mothers have secondary education have about 33% lower risk [odds-ratio=0.67; 95% CI=0.51-0.87] of suffering from malaria infection compared to those whose mothers did not attend school. There is no significant difference between children whose mothers attended only primary school and those whose mothers did not attend school considering the prevalence of malaria infection among children. Children who slept under a mosquito net have 14% lower risk [odds-ratio=0.86; 95% CI=0.74-0.99] of suffering from malaria infection compared to their counterparts who did not sleep under a mosquito net.

Table 5 – Factors associated with malaria infection among children aged less than 5 years in the DRC: Findings from logistic regression

|  | Odds Ratio | P-value | 95% Confidence interval | |
| --- | --- | --- | --- | --- |
| **Child' sex** |  |  |  |  |
| Male | Reference |  |  |  |
| Female | 0.962 | 0.578 | 0.841 | 1.101 |
| **Child' age** |  |  |  |  |
| 6-11 months | Reference |  |  |  |
| 12-23 | 1.713 | <0.001 | 1.245 | 2.357 |
| 24-35 | 2.675 | <0.001 | 2.020 | 3.543 |
| 36-47 | 3.356 | <0.001 | 2.526 | 4.459 |
| 48-59 | 3.417 | <0.001 | 2.527 | 4.622 |
| **Mother's education** |  |  |  |  |
| No education | Reference |  |  |  |
| Primary | 0.965 | 0.773 | 0.754 | 1.233 |
| Secondary&+ | 0.669 | 0.003 | 0.513 | 0.874 |
| DKN | 0.892 | 0.447 | 0.665 | 1.198 |
| **Slept under mosquito net** |  |  |  |  |
| No | Reference |  |  |  |
| Yes | 0.857 | 0.044 | 0.738 | 0.996 |
| **Sex of head of household** |  |  |  |  |
| Male | Reference |  |  |  |
| Female | 0.841 | 0.083 | 0.692 | 1.023 |
| **Age of head of household** |  |  |  |  |
| Less than 24 years old | Reference |  |  |  |
| 25-34 | 0.878 | 0.394 | 0.652 | 1.184 |
| 35-44 | 0.871 | 0.392 | 0.633 | 1.196 |
| 45-64 | 0.980 | 0.897 | 0.719 | 1.335 |
| 65&+ | 0.779 | 0.248 | 0.510 | 1.190 |
| **Household Wealth Index** |  |  |  |  |
| Poorest | Reference |  |  |  |
| Poorer | 1.201 | 0.120 | 0.953 | 1.512 |
| Middle | 1.000 | 0.997 | 0.765 | 1.309 |
| Richer | 0.692 | 0.028 | 0.498 | 0.962 |
| Richest | 0.187 | <0.001 | 0.095 | 0.369 |
| **Type of place of residence** |  |  |  |  |
| Capital, large city | Reference |  |  |  |
| Small cities &. Town | 1.084 | 0.810 | 0.562 | 2.090 |
| Rural | 0.808 | 0.510 | 0.429 | 1.524 |
| **Province of residence** |  |  |  |  |
| Kinshasa | Reference |  |  |  |
| Kwango | 0.106 | <0.001 | 0.035 | 0.318 |
| Kwilu | 0.096 | <0.001 | 0.035 | 0.268 |
| Mai-ndombe | 0.399 | 0.081 | 0.142 | 1.120 |
| Kongo Central | 0.447 | 0.084 | 0.179 | 1.114 |
| Equateur | 0.189 | 0.039 | 0.039 | 0.923 |
| Mongala | 0.256 | 0.015 | 0.085 | 0.769 |
| Nord-Ubangi | 0.464 | 0.119 | 0.176 | 1.218 |
| Sud-Ubangi | 0.211 | <0.001 | 0.082 | 0.542 |
| Tshuapa | 0.190 | <0.001 | 0.069 | 0.524 |
| Kasai | 0.339 | 0.080 | 0.101 | 1.137 |
| Kasai-Central | 0.634 | 0.319 | 0.259 | 1.555 |
| Kasai-Oriental | 0.493 | 0.069 | 0.230 | 1.056 |
| Lomami | 0.614 | 0.299 | 0.245 | 1.543 |
| Sankuru | 0.175 | 0.002 | 0.057 | 0.532 |
| Haut-katanga | 0.633 | 0.319 | 0.257 | 1.558 |
| Haut-lomami | 0.394 | 0.094 | 0.132 | 1.174 |
| Lualaba | 0.837 | 0.764 | 0.261 | 2.681 |
| Tanganyka | 0.915 | 0.865 | 0.329 | 2.543 |
| Maniema | 0.441 | 0.087 | 0.173 | 1.128 |
| Nord-Kivu | 0.053 | <0.001 | 0.018 | 0.157 |
| Bas-Uele | 1.028 | 0.953 | 0.407 | 2.598 |
| Haut-Uele | 0.760 | 0.635 | 0.244 | 2.369 |
| Ituri | 0.512 | 0.199 | 0.184 | 1.423 |
| Tshopo | 0.344 | 0.029 | 0.132 | 0.897 |
| Sud-Kivu | 0.092 | <0.001 | 0.033 | 0.258 |
| _cons | 0.68647 | 0.377 | 0.2973776 | 1.584656 |

After controlling for other variables, the risk of suffering from malaria infection among children aged less than five year is lower in all provinces compared to Kinshasa. However, the difference is statistically significant only in the following 10 provinces: Kwango, Kwilu, Equateur, Mongala, Sud-Ubangi, Tshuapa, Sankuru, Nord Kivu, Tshopo and Nord Kivu (P-value: <0.05).

**Discussion**

This study aimed to identify predictors of malaria infection among children aged under 5 years in the DRC and describe socioeconomic profile of children suffering from malaria infection. The discussion is organized around three points: complexity of findings, complementarity between methodological approaches and policy implications. Box 2 summarizes key findings and reports those that are consistent with the literature.

Box 2 – Summary of key findings

| **Variables** | **Chi-square (Bivariate)** | **Logistic regression** | **CHAID** | **Consistency with literature** |
| --- | --- | --- | --- | --- |
| Child’ sex | Not significant | Not significant | Not significant | Yes |
| Child’s age | Significant | Significant | Significant | Yes |
| ITN used the night before the survey | Significant | Significant | Not significant | Depend on method |
| Place of residence | Significant | Not significant | Significant | Depend on method |
| Mother’s education | Significant | Significant | Significant | Yes |
| Province | Significant | Significant | Significant | Yes |
| Wealth index | Significant | Significant | Significant | Yes |

Of the ten variables analyzed, four were statistically associated with the prevalence of malaria infection in bivariate analysis and multivariate analysis (CHAID and logistic regression): child’s age, mother’ education, province and wealth index. These findings are consistent with previous studies ^[8-9, 13-25]^. The risk of suffering from malaria infection among children aged less than five years increase with child age. Two hypotheses, which we were not able to test in this study, may explain this finding. First, younger children may be protected from malaria because of the antibodies they acquire from their mothers during pregnancy and during breastfeeding ^[33]^. Last, younger children in some sub-Saharan Africa, including the DRC share a bed with their mothers and are more likely to be covered properly with a blanket or mosquito net than older children ^[34-36].^ Figure 3 shows proportion of under-five children who slept under a mosquito net the night preceding the study by age in DRC.

[Figure 3, about here]

Findings also show that the higher the level of mother’s level of education, the lower the prevalence of malaria among under-five children. Previous studies reported that mothers with higher levels of education were more knowledgeable about malaria prevention and signs and were therefore more proactive and reactive regarding prevention than mothers with lower levels of education ^[34,37-38]^. Higher mother’s education serves as a protective factor against infectious diseases such as malaria because higher level of education is associated with more knowledgeable about malaria prevention and signs, and more proactive and reactive with regard to prevention than mothers with lower levels of education ^[5, 20, 34, 38-40]^. In 2018, proportion of children aged less than five years who slept under an ITN was higher (more than 60%) among children of the most educated mothers (secondary education or higher) compared to their counterpart whose mothers did not reach that level (36% and 46% respectively for children whose mothers did not attend school or attended only primary school) ^[39]^. The results of this study also show that malaria cases were less prevalent among children from richest households than those from poorest households. People with higher wealth quintile are more likely to live improved houses and more likely to be educated and have better access to knowledge about steps to prevent malaria infection. They were also more likely to be able to afford ITNs and be able to use them correctly as well as be able to afford insecticides used for indoor spraying ^[5, 20, 38-40]^. Data from the 2018 Multiple Indicators Cluster Survey, reported that the proportion of under five children who slept under an ITN varied from 35% in poorest household to 69% in the richest household ^[39]^.

Provincial differences in malaria prevalence among under five children could be explained by environmental and ecological factors, including the distances from household and the nearest water body, altitudes, temperature and rainfall ^[6, 8-9, 19-20]^. The 2013 DHS as well as the 2018 MICS data report variation of the use of ITN among children aged less than 5 years by province in the Democratic Republic of Congo ^[26, 39]^.

Surprisingly, while bivariate analysis and logistic regression models report low prevalence of malaria infection among children who slept under an ITN lower (odds ratio [OR] = 0.86; 95% CI -0.74 -0.99; p = 0.04) compared to those who were not protected, the variable “slept under mosquito net” is not statistically associated with the prevalence of malaria if one considers findings from CHAID model. It is likely that this effect has been captured by other variables such age, mother’s education, household’ wealth index, place of residence and province of residence which are associated with the use of ITN by children aged less than 5 years and with of the prevalence of malaria among under five children. In previous study, Ferrari ^[16]^ found that the effect of mosquito nets was not significant in the lower transmission strata in the DRC. In parallel the study reported that children aged less than two years were more likely to sleep under a mosquito net compared to others ^[16]^.

The study shows also that effect of place of residence was not statistically significant in the logistic regression model. The CHAID model reported significant differences between children living in urban areas and those living in rural contexts, particularly among children living in : (1) Kwango, Nord Kivu, Kwango, Lualaba and Bas Uele; and (2) in Equateur, Mongala, Tshuapa, Sud Kivu and Kinshasa aged 36-59. This difference could be explained by the fact that logistic regression does not automatically detect interactions between independent variables or the segments in which the model is statistically significant.

Comparison of findings by method of analysis shows also that the province of residence (spatial location) is the most important predictor of malaria prevalence among under five children in DRC ^[8-9, 13-20, 37]^. The effect of other variables such as child’s age, place of residence, maternal education or wealth index depends on the province. For instance, maternal education is only significant for children aged 6-11 months and 12-35 months living in Ituri, Kasai-Central, Haut-Uele, Lomami, Nord-Ubangi, Maniema provinces. The findings show also that children belonging to the same socioeconomic category (e.g. age, place of residence, wealth index) might belong to different risk groups (high or less) depending on their region (province) of residence. These findings support results from Ferrari ^[16]^, which revealed that predictors of child malaria varied by strata (malaria high transmission zone versus malaria low transmission zone). Furthermore, these findings suggest that the prevalence of malaria infection is driven by interaction between environmental factors, socioeconomic characteristics and probably differences in the implementation of malaria programs across the country. Box 3 summarizes key findings and recommendations.

Box 3 – Summary of key findings and recommendations

| **Key Findings** | **Recommendations** |
| --- | --- |
| Prevalence of malaria infection is driven by interaction between environmental factors, socioeconomic characteristics | Malaria program should become “National Multisectoral Malaria Program” involving the ministry of health, agricultural, Education, Urbanization and Habitat, Rural Development, Social and Humanitarian affairs, Interior affairs, Gender and family, Environment (Figure 4). |
| Existence of high-risk groups for malaria in the majority of provinces | Universal coverage of insecticide-treated nets and house improvement should be implemented in all provinces because they are the most cost-eﬃcient intervention to reduce both burden and transmission, irrespective of the ecology within a setting ^[9, 18, 20]^.  Include malaria education in school curriculum as part of the stand-alone “Family Life Course” implemented in DRC schools because malaria is a public health problem with a prevalence of 25% among under-five children ^[41]^.  Integrate malaria prevention and care into workplace policies and use campaigns to raise awareness among employees ^[41]^. |
| Spatial variation of malaria: malaria prevalence is high in some provinces. | Promote province-based implementation studies on malaria as well as malaria interventions. In high endemic clusters (prevalence above 40%), insecticide-treated nets could be associated with seasonal malaria chemoprevention with seasonal malaria chemoprevention or indoor residual spraying ^[43-44]^. |
| High prevalence of malaria among children aged 24 months and above. | Increase vitamin A and zinc supplementation among under-five children as part of immunization ^[37]^. |
| High prevalence of malaria among children of mothers with low education and/or living in poorest households and/or living in rural areas. | Promote the community engagement strategy for malaria prevention and treatment; and share malaria prevention information on social media ^[41].^ |

Transforming the current malaria program to a “National Multisectoral Malaria Program” (NMMP) involving the ministry of health, agricultural, Education, Urbanization and Habitat, Rural Development, Social and Humanitarian affairs, Interior affairs, Gender and family, Environment (Figure 4) will strengthen the fight again malaria. This institution should involve also key stakeholders working on malaria and other related programs, including members of the civil society organizations (CSO), non-governmental organizations (NGO), academia.

[Figure 4 about here]

The current national program will play the role of Technical Secretariat. Such institution will be consistent with the UNDP and MRB multisectoral framework for malaria ^[5]^. It will design the national malaria policy and advise the government.

*Study limitations*

This study suffers from two methodological limitations, which do not affect its quality. First, the CHAID model does not consider the hierarchical structure of the DHS data, which might influence the overall prevalence of malaria infection. However, CHAID does allow automatic detection of segments in which the prevalence of malaria infection is similar and addresses the failure to incorporate non-monotonic relationships in logistic regression. Furthermore, CHAID is a diagnostic technique partitioning the data set into several segments. This differ by the misclassification error of logistic regression model ^[31]^. Because of the heterogeneity of the data, segment wise prediction models (CHAID) is more advantageous than the logistic global model.

Second the study did not control for the seasonality of malaria transmission. DRC is crossed by the equator, with rainy and dry seasons varying across the country by province, district and/or health zone within the same province. Unfortunately, DHS data did not collect information about the season.

Missing values were treated as a separate category. For instance, the “Do not know” category for maternal education included children whose mother’s education was missing.

**Conclusion**

In summary, findings from this study could be used for designing malaria target interventions in DRC. They show heterogeneity in malaria burden among under-five children in DRC. Consistent with findings from previous studies, four of the nine variables included in multivariate models (logistic regression and CHAID) were statistically associated with the prevalence of malaria infection: child’s age, mother’ education, province and wealth index. Furthermore, findings from the CHAID model reveal that predictors of malaria infection vary by province. Within each province, child’s age is the common predictor of malaria infection. Other predictors include place of residence, wealth index and maternal education. These findings suggest also that the prevalence of malaria infection is driven by interaction between environmental factors, socioeconomic characteristics and probably difference in the implementation of malaria programs across the country. The most-at-risk groups for malaria in one province might not be necessary the ones at the greater risk in other provinces. Therefore, designing province and multisectoral interventions could be the most effective strategies to achieve zero malaria infection in DRC. Some of the key interventions are outlined in the *World Malaria Report 2019* ^[42]^ and include investments in malaria programs and research; malaria prevention; diagnostic testing a treatment; and malaria surveillance systems.

Due to the multiplicity of factors that are linked to malaria transmission, it is important that various actions that directly and indirectly affect malaria prevention policy are designed and operate in synergy, via a multi-sectorial malaria prevention policy and national program.

**Bibliography**

[1] United Nations. (2015). The Millennium Development Goals Report. New York: United Nations.

[2] African Union Commission. (2013). United Nations Economic Commission for Africa; UNFPA. Addis Ababa Declaration on Population and Development in Africa beyond 2014. Addis Ababa, Ethiopia: African Union Commission.

[3] WHO. (2015). The Global Technical Strategy for Malaria 2016-2030. Geneva: WHO.

[4] WHO. (2017). World malaria report. Geneva: WHO.

[5] United Nations Development Programme (UNDP) and Roll Back Malaria Partnership (RBM). (2013). Multisectoral Action Framework for Malaria. New York: UNDP and RBM.

[6] Tanner, M., Greenwood, B., Whitty, C. M., Ansah, E., Price, R., Dondorp, A., . . . Osier, F. (2015). Malaria eradication and elimination: views on how to translate a vision into reality. BMC Medicine, DOI 10.1186/s12916-015-0384-6.

[7] World Health Organization Regional Office for Africa (2016). Framework for implementing the global technical strategy for malaria 2016–2030 in the african region. Addis Ababa, Federal Democratic Republic of Ethiopia: World Health Organization Regional Office for Africa.

[8] WHO. (2018). World malaria report. Geneva: WHO.

[9] PNLP. (2017). Evaluation de l’impact des interventions de lutte contre le paludisme sur la mortalité toutes causes confondues chez les enfants de moins de cinq ans en République Démocratique du Congo de 2005 à 2015. Kinshasa: PNLP.

[10] PNLP. (2016). Rapport d' activités 2016: Kinshasa: PNLP.

[11] Lechthaler, F.; Matthys, B.; Lechthaler-Felber, G.; Losimba, J.L.; Muhindo, H.M; Matangila, J.R.; Mutombo, M.M. ; Ruckstuh, L.; Barczyk, J.; Shargie, E.; Prytherch, H.; Lengeler, C. (2019) Trends in reported malaria cases and the effects of malaria control in the Democratic Republic of the Congo. PLoS ONE 14(7): e0219853

[12] Ferrari, G.; Ntuku, H.M.; Schmidlin, S.; Diboulo, E.; Tshefu, A.K.; Lengeler, C. (2016). A malaria risk map of Kinshasa, Democratic Republic of Congo. Malaria Journal. DOI 10.1186/s12936-015-1074-8.

[13] Mulangu, A.M.; Mukuku, O.; Nzeba, K.T.; Kawawa, E.S.; Mukomena, E.; Tshikwej, D.N.; Numbi, O.L.; Kakoma, JB.; Okitotsho, S.W.; Van Geertruyden, JP; Lutumba, P. (2018). Severe malaria and death risk factors among children under 5 years at Jason Sendwe Hospital in Democratic Republic of Congo. Pan African Medical Journal. doi:10.11604/pamj.2018.29.184.15235.

[14] Mutombo, A; Ndua, J; Mukuku, O; Tshibanda, C; Mukomena, E; Wembonyama, S.(2017) Severe malaria and risk factors associated to the lethality in children admitted in Jason Sendwe Hospital, Democratic Republic of the Congo. Tropical Medicine & International Health:307.

[15] Ilunga-Ilunga, F.; Levêque, A.; Donnen, P.; M. Dramaix (2015). Caractéristiques des ménages des enfants hospitalisés pour paludisme grave et facteurs associés à la létalité palustre à Kinshasa (République démocratique du Congo). Médecine et Santé Tropicales. 10.1684/mst.2014.0416.

[16] Ferrari, G., Ntuku, H., Ross, A., Schmidlin, S., Kalemwa, D., Tshefu, A. K., & Lengeler, C. (2016). Identifying risk factors for Plasmodium infection and anaemia in Kinshasa, Democratic Republic of Congo. Malaria Journal, DOI 10.1186/s12936-016-1412-5.

[17] Ngatu, R.N.; Kanbara, S.; Renzaho, A.; Wumba, R.; Mbelambela, P.E.; Muchanga, M.S.J.; Muzembo,A.B.; Kabamba, L.N.; Nattadech, C.; Suzuki, T.; Luboya, O.N.; Wada, K.; Ikeda, M.; Nojima, S.; Sugishita, T.; Ikeda, S. (2019) Environmental and sociodemographic factors associated with household malaria burden in the Congo. Malaria Journal. <https://doi.org/10.1186/s12936-019-2679-0>.

[18] Levitz, L., Janko, M., Kashamuka, M., Thwai, K., Likwela, J., Tshefu, A., . . . Meshnick, S. (2018). Efect of individual and community-level bed net usage on malaria prevalence among under-fves in the Democratic Republic of Congo. Malaria Journal, <https://doi.org/10.1186/s12936-018-2183-y>.

[19] World Health Organization and the Commission for Technical Co-operation in Africa South of the Sahara. (1951). Malaria Conference in Equatorial Africa. Technical Report Serie. Geneva: WHO.

[20] Ye, Y., Hoshen, M., Louis, V., Seraphin, S., Traore, I., & Sauerborn, R. (2006). Housing conditions and Plasmodium Falciparum infection: protective effect of iron – sheet roofed houses. Malaria journal, doi:10.1186/1475-2875-5-8.

[21] Balami, A., Said, S., Zulkefli, N. M., Bachok, N., & Audu, B. (2019). Effects of a health educational intervention on malaria knowledge, motivation, and beha0vioural skills: a randomized controlled trial. Malaria Journal , <https://doi.org/10.1186/s12936-019-2676-3>.

[22] Osterbauer, B., Kapisi, J., Bigira, V., Mwangwa, F., Kinara, S., Kamya, M., & Dorsey, G. (2012). Factors associated with malaria parasitaemia, malnutrition, and anaemia among HIV-exposed and unexposed Ugandan infants: a cross-sectional survey. ;11:432. Malaria Journal, doi:10.1186/1475-2875-11-432.

[23] Rowe, A., Rowe, S., Snow, R., Korenromp, E., Schellenberg, J., Stein, C., . . . Steketee, R. (2006). The burden of malaria mortality among African children in the year 2000. International journal of epidemiology, 691–704.

[24] Wanzira, H., Katamba, H., Okullo, A., Agaba, B., Kasule, M., & Rubahika, D. (2017). Factors associated with malaria parasitaemia among children under 5 years in Uganda: a secondary data analysis of the 2014 Malaria Indicator Survey dataset. Malaria Journal, DOI 10.1186/s12936-017-1847-3.

[25] Zgambo, M., Mbakaya, B., & Kalembo, F. (2017). Prevalence and factors associated with malaria parasitaemia in children under the age of five years in Malawi: A comparison study of the 2012 and 2014 Malaria Indicator Surveys (MISs). PLoS ONE, e0175537.

[26] Ministère de Plan et Suivi de la Revolution de la modernité; Ministère de la Santé Publique (MSP) et ICF International. (2014). Enquête Démographique et de Santé en République Démocratique du Congo 2013-2014. MPSMRM, MSP et ICF International: Rockville, Maryland, USA.

[27] Emina, J., Madise, N., Kuepie, M., Zulu, E., & Ye, Y. (2013). "Identifying HIV most-at-risk groups in Malawi for targeted interventions. A classification tree model." . BMJ open, e002459.

[28] Shah, J., Emina, J., Eckrt, E., & Ye, Y. (2014). Prompt access to effective malaria treatment among children under five in sub-Saharan Africa: a multi-country analysis of national household survey data. Malaria Journal, DOI 10.1186/s12936-015-0844-7.

[29] Kass, G. (1980). Anexploratory technique forinvestigating large quantities of categorical data. Applied Statistics, 29, 119-127.

[30] Kitsantas, P., Hollander, M., & Li, L. (2006 ). Using classification trees to assess low birth weight outcomes. Artif Intell Med, 275–289.

[31] Antipov, E., & Pokryshevskaya, E. (2010 ). Applying CHAID for logistic regression diagnostics and classification accuracy improvement. Journal of Targeting, Measurement and Analysis for Marketing, 109 – 117.

[32] Kleinbaum, D. G. (1994). Logistic Regression: A Self-Learning Text. New York: Springer-Verlag.

[33] Centre for Disease Control and Prevention (2010). Human Factors and Malaria Centres for Disease Control and Prevention, Centre for Disease Control and Prevention, Atlanta, Ga, USA.

[34] Chilanga, E.; Collin‑Vézina, D.; MacIntosh, H.; Mitchell, C. & Cherney, K. (2020). Prevalence and determinants of malaria infection among children of local farmers in Central Malawi. Malaria Journal. <https://doi.org/10.1186/s12936-020-03382-7>.

[35] Eisele, T.P; Keating, J; Littrell, M.; Larsen, D.; & Macintyre, K. (2009). Assessment of insecticide-treated bednet use among children and pregnant women across 15 countries using standardized national surveys. American Journal of Tropical Medicine & Hygiene: 209–214.

[36] Mugisha, F; Arinaitwe, J (2003). Sleeping arrangements and mosquito net use among under-five: results from the Uganda Demographic and Health Survey. Malaria Journal. 2:40.

[37] Zeba, A., Sorgho, H., Rouamba, N., Zongo, I., Rouamba, J., Guiguemdé, R., . . . Ouedraogo, J.-B. (2008). Major reduction of malaria morbidity with combined vitamin A and zinc supplementation in young children in Burkina Faso: a randomized double-blind trial. Nutrition Journal, doi:10.1186/1475-2891-7-7.

# [38] Oyekale, A.S. (2015) Assessment of Malawian mothers’ malaria knowledge, healthcare preferences and timeliness of seeking fever treatments for children under five. International Journal of Environmental Research and Public Health: 521–540.

[39] Institut National de la Statistique (2019) Enquête par grappes à indicateurs multiples, 2017-2018, rapport de résultats de l’enquête. Kinshasa, République Démocratique du Congo.

# [40] Morakinyo, O.M.; Folusho M. Balogun, F.M; Fagbamigbe, A.F. (2018) Housing type and risk of malaria among under-five children in Nigeria: evidence from the malaria indicator survey. <https://doi.org/10.1186/s12936-018-2463-6>.

[41] African Union Commission with the RBM Partnership to End Malaria (2020).Zero Malaria Starts with Me Toolkit.

[42] Walker, P., Griﬃn, J., Ferguson, N. M., & Ghani, A. (2016). Estimating the most eﬃcient allocation of interventions to achieve reductions in Plasmodium falciparum malaria burden and transmission in Africa: a modelling study. Lancet Global Health, 10.1016/ S2214-109X(16)30073-0.

[43] Quakyi, I., Tornyigah, B., Houze, P., Kusi, K., Coleman, N., Escriou, G., . . . Ndam, N. (2019). High uptake of Intermittent Preventive Treatment of malaria in pregnancy is associated with improved birth weight among pregnant women in Ghana. Scientific Reports , <https://doi.org/10.1038/s41598-019-55046-5>.

[44] Tinashe, A., Tizifa, T., Kabaghe, A., McCann, R., van den Berg, H., Van Vugt, M., & Phiri, K. (2018). Prevention Efforts for Malaria. Current Tropical Medicine Reports, 41–50.

World Health Organization (2019). World malaria report 2019. Geneva: World Health Organization.

### **List of abbreviations**

| CHAID | Chi-square automatic interaction detector |
| --- | --- |
| CSO | Civil Society Organization |
| DHS | Demographic and Health Survey |
| DRC | Democratic Republic of Congo |
| IPTp | Intermittent Preventive Treatment during Pregnancy |
| ITNs | Insecticide-Treated Nets |
| MDG | Millennium Development Goals |
| MDG | Millennium Development Goal |
| MPSMRM | Ministère de Plan et de Suivi de la mise en oeuvre de la révolution de la modernité |
| MSP | Ministère de Santé Publique/ Ministry of Public Health |
| NGO | Non-Governmental Organization |
| NMMP | National Multisectoral Malaria Program |
| PNLP | Programme National de Lutte contre le Paludisme/ national malaria control program |
| RBM | Roll Back Malaria |
| SDG | Sustainable Development Goal |
| UNDP | United Nations Development Programme |
| WHO | World Health Organization |

**Declarations**

*Ethics approval and consent to participate*

The DHS questionnaire and the testing protocol undergo a host country ethical review as well as an ethical review at ICF Macro. Furthermore, participation in individual survey and in malaria testing is voluntary. Parents should sign the consent form before interview and before children’ blood collection.

*Availability of data and material*
This study is based on the DRC 2013 Demographic and Health Surveys. These data are available on https://www.dhsprogram.com. Access to the file required authorization from the DHS program (https://www.dhsprogram.com). The website depicts the process.

*Competing interests*: None
*Funding*
This research did not receive any specific funding. Yazoumé Yé (YY) is M&E Infectious Disease, ICF International, USA employee, the institution did not have any role in the study design, data collection and analysis, decision to publish, or preparation of the manuscript. The specific roles of these authors are articulated in the ‘author contributions’ section. The contribution of YY was not part of his work time with M&E Infectious Disease, ICF International.

*Acknowledgements*

The authors thank Macro International for providing free access to the 2013 datasets for the Demographic and Health Survey. They also thank Michelle Tsagli for her comments on the earlier version of the paper.

1. All malaria cases are not reported in health facilities.
